# Schizophrenia, Not a Psychotic Disorder: Bleuler Revisited

**DOI:** 10.3389/fpsyt.2019.00328

**Published:** 2019-05-10

**Authors:** Alexandre Andrade Loch

**Affiliations:** ^1^Laboratory of Neuroscience (LIM 27), Institute of Psychiatry, University of São Paulo, São Paulo, Brazil; ^2^Instituto Nacional de Biomarcadores em Neuropsiquiatria (INBION), Conselho Nacional de Desenvolvimento Cientifico e Tecnológico, São Paulo, Brazil

**Keywords:** prodromal psychosis, psychosis continuum, ultra high risk, negative symptoms, neurodevelopment hypothesis

## Abstract

Current diagnostic criteria delineate schizophrenia as a discrete entity essentially defined by positive symptoms. However, the role of positive symptoms in psychiatry is being questioned. There is compelling evidence that psychotic manifestations are expressed in the population in a continuum of varying degrees of severity, ranging from normality to full-blown psychosis. In most cases, these phenomena do not persist, but they constitute risk factors for psychiatric disorders in general. Psychotic symptoms are also present in most non-psychotic psychiatric diagnoses, being a marker of severity. Research revealed that hallucinations and delusions appear to have distinct, independent biological underpinnings—in the general population, in psychotic, and in non-psychotic disorders as well. On the other hand, negative symptoms were seen to be far more restricted to schizophrenia, have other underlying pathophysiology than positive symptoms, predict outcome and treatment response in schizophrenia, and start before the first psychotic outbreak. The current work discusses the concept of schizophrenia, suggesting that a greater emphasis should be put on cases where psychotic symptoms emerge in a premorbid subtly increasing negative/cognitive symptoms background. In those cases, psychosis would have a different course and outcome while psychosis occurring in the absence of such background deterioration would be more benign—probably having no, or a milder, underlying degenerative process. This reformulation should better drive psychopathological classification, face positive symptoms as epiphenomenon of the schizophrenia process, and dishevel stigma from schizophrenia and from delusions and hallucinations.

## Introduction

According to the *Diagnostic and Statistical Manual of Mental Disorders, Fifth Edition* (*DSM-5*) (1), schizophrenia is essentially defined by positive symptoms. Diagnosis is made upon a 1-month presence of either hallucinations, delusions, or disorganized speech plus another criterion A symptom—which adds grossly disorganized/catatonic behavior and negative symptoms to the three aforementioned symptoms. However, schizophrenia’s diagnosis hasn’t always been like that.

Schizophrenia was first described as *dementia praecox* and popularized by Emil Kraepelin in 1893 (2). Guided by a natural disease unit idea, Kraepelin assigned those with psychotic symptoms and progressive cognitive decline into the diagnosis. On the other hand, manic-depressive illness would constitute a distinct psychotic category based on different course and outcome (3). Following the medical model of that time, Kraepelin believed that mental disorders would be manifested in discrete entities, with clear-cut boundaries between disorders, and between normality and pathology. Nevertheless, Kraepelin himself later began to question whether clinicians could accurately assign patients to his “natural disease units” (4). Eugen Bleuler then was the first to coin the term *schizophrenia* in 1908 (3, 5). Contrasting Kraepelin, Bleuler conceptualized the disorder in a dimensional framework, assuming a continuity between psychosis and normality (6). More importantly, he identified ambivalence, autism, affective incongruity, and association disturbances—the “four A’s”—as the basic symptoms, relegating hallucinations and delusions to “secondary symptoms” (7). The categorical and dimensional approaches coexisted for some decades until the neo-Kraepelinean era came with the *DSM-III* (8). The descriptive and categorical approach to psychosis and other psychiatric diagnoses was resumed, a view that remains in the present day with the *DSM-5* (1, 9).

However, recent findings question again the neo-Kraepelinean paradigm. Epidemiological and biological research show that mental disorders’ manifestations and pathophysiology do not obey the boundaries of current diagnostic categories. The categorical versus dimensional discussion returned to the spotlight, and the role of symptoms in psychiatry is being questioned (10). Hence, the importance of positive symptoms to the diagnosis of schizophrenia must also be reconsidered.

## What is Psychosis? About Symptoms and Disorders

Regarding psychosis too, not always has it been conceptualized as we know psychosis nowadays. The term was first introduced in the psychiatric literature by Canstatt in 1841 as a synonym for *psychic neurosis* (11). Psychosis was then a broad concept that simply translated a psychic manifestation of brain’s disease. This was the first detachment of psychosis from the neurosis construct. The notion of psychosis was then further developed into endogenous and exogenous psychosis in the first half of the 20th century. The first depicted disorders where an existing somatic cause was present but not identifiable, whereas the latter was caused by extraneous influence—somatic or psychic (12). The discovery of new pathological causes of diseases and development in the field of neuropathology strengthened the idea of psychosis as having a biological basis, leading to the constriction of the term *neurosis* to purely psychogenic disorders (12). The psychosis–neurosis dichotomy was definitely consolidated by Freud’s psychoanalysis, designating as neurotic those entities caused by psychological conflicts originating in childhood. Neurosis would be amenable to psychoanalytical intervention, whereas psychosis would not—an idea that Freud himself would refute later on his life. Nevertheless, with the increasing discovery of the biological underpinnings of mental illnesses—including depression, OCD, and anxiety, among other typical “neuroses”—the neurosis–psychosis duality slowly started to be abandoned. In the *DSM-II*, psychosis was unspecifically defined and neurosis was subsumed under the heading of “hysterical neurosis,” embracing conversion and dissociative disorders. From the *DSM-III* on, neurosis vanishes from classificatory categories and psychosis is limited to its adjectival form *psychotic*, which remains until today (8).

As such, psychosis in current psychiatric classification systems is restricted to psychotic symptoms—comprising hallucinations and delusions—and psychotic disorders, syndromes where psychotic symptoms dominate. Therefore, psychosis is characterized by what is grossly observed—likewise the other diagnostic categories in *DSM-5*. More subtle notions of psychosis like Bleuler’s four A’s and the phenomenological approach are abandoned.

Using a phenomenic rather than a phenomenological approach, for instance, has some advantages for research, communication, and legal purposes. Nevertheless, there are some serious problems inherent to this method.

Taking psychotic disorders as an example, the first step for characterizing such diagnosis would be the accumulation and/or persistence of observable psychotic symptoms over some time—*DSM-5* “A” syndromal feature of a mental disorder. However, how many psychotic symptoms must one have to be diagnosed as having a disorder? And how long should they last? Also, strictly speaking, if it does not imply in a disorder/disease, it should be named as a manifestation or phenomenon, e.g., and not a symptom. Syndromes in psychiatry—according to the current phenomenic understanding—are characterized in most cases by quantitative changes of normal phenomena, and this feature intrinsically requires the arbitration of a cutoff point. But that’s not all, and here is where it gets even more complicated. Further criteria to be added to the syndromal one involves disability, distress, risk of suffering death or important loss of freedom, not being culturally sanctioned, and other arbitrary judgments (13). The increasing addition of more arbitrariness to diagnostic criteria eroded the distinction between psychopathology and normal psychological phenomena. It turns out that, instead of psychosis, or a psychotic disease, the term *psychotic disorder* “may be preferable insofar as it emphasizes that these conditions are not purely ‘mental’, and that the line between ‘psychiatric disorder’ and ‘other medical disorder’ is not a sharp one” (13).

According to some authors, schizophrenia would represent 44% of today’s psychotic disorder diagnosis (14). However, as stated earlier, current categories only take into account the phenomenic aspect of mental disturbances, not considering all the available biological findings underlying them. In light of these ideas, this article will now properly step into the discussion of the importance of positive symptoms in the definition of schizophrenia. To facilitate the reading, we will use the current definition of psychosis as referring to hallucinations and delusions. Psychotic *experiences* and *phenomena* will refer to both the clinical and subclinical spectrum of delusion and hallucination’s manifestations—e.g., illusions, pseudo-hallucinations, true hallucinations. On the other hand, when used, psychotic *symptoms* will refer only to clinically relevant (true) hallucinations and delusions.

## Psychotic Manifestations Beyond Psychotic Disorders

It is generally acknowledged that psychotic phenomena do not occur on an all-or-nothing basis. They are expressed in the population in a *continuum* of varying degrees of intensity and severity, ranging from normality to full-blown psychotic disorders. A meta-analytic study reported a median prevalence of 5–8% of psychotic experiences in the general population, while only 3% of individuals had a psychotic disorder diagnosis (15). Hence, psychotic symptoms are ubiquitously distributed in the general population, (1) not being restricted to the psychosis disorders diagnoses, and (2) not being restricted even to psychiatric diagnoses at large.

Concerning the first issue, previous studies reported on the presence of psychotic symptoms in several psychiatric disorders (16, 17). In Alzheimer’s disease, psychotic symptoms are regarded as markers of poor prognosis, being associated with more rapid cognitive decline, more severe cognitive impairment (18), institutionalization, and mortality (19). Psychotic symptoms are usually related to low functioning and disability (20) and worse cognitive performance in bipolar disorder (BD) patients (21). Psychotic phenomena were also found to occur in major depressive disorders and anxiety disorders (22, 23), being related to a more severe condition in such cases (24). Obsessive–compulsive disorder cases with psychotic features have more often a deteriorative course compared to those without psychotic features (25).

Besides being a marker of severity in most non-psychosis diagnoses, psychotic symptoms are also observed in otherwise healthy individuals (17). In the United States National Comorbidity Survey, 28% of individuals endorsed a psychosis screening item while only 0.7% were regarded as having a clinician-defined psychotic disorder (26). In the Dunedin cohort, 25% of the sample reported at least one lifetime delusional or hallucinatory experience that was unrelated to drug use or physical illness, but only 3.7% fulfilled criteria for schizophreniform disorder (27). In the Dutch NEMESIS, 7076 individuals were interviewed with the Composite International Diagnostic Interview (CIDI); 17.5% scored at least one lifetime psychosis item but only 2.1% received a psychotic disorder diagnosis (16). Hence, only a small part of the total phenotypic continuum of psychosis is represented by clinical cases. A systematic review of such studies describes a prevalence of 8% of psychotic experiences, while psychotic symptoms and psychotic disorder occur in 4% and 3% of the general population, respectively (28).

Although the majority of these phenomena found in the community do not persist (29), they are regarded as risk factors for forthcoming psychotic disorders and general psychiatric diagnoses (30). An 8-year follow-up of 914 adolescents observed that self-reported auditory hallucinations were markers of future non-psychotic diagnosis (31). In the Netherlands, 1912 adolescents were followed up for 2 years. Auditory hallucinations were displayed by 5% of them at baseline, being related to increased levels of depression and general psychopathology at follow-up. Most of these phenomena—two-thirds of them—discontinued, but hallucination’s persistence was associated with increased risk for follow-up delusional ideation (32). In an Irish study enrolling 1,131 adolescents, participants reporting a psychotic experience had poorer performance than those who did not report such an experience. Furthermore, participants with psychiatric disorders who reported psychotic experiences had significantly poorer functioning compared to adolescents with psychiatric disorders not reporting psychotic experiences—replicating the above-discussed aspect of psychosis as a marker of illness severity (33).

However, results concerning functioning and psychopathology in healthy individuals with psychotic experiences are not unambiguous. A study of 101 healthy individuals with auditory verbal hallucinations showed that their cognitive profile was largely similar to that of healthy controls without hallucinations (34). Healthy individuals with hallucinations only had slightly—but significantly—lower scores on level of verbal intellectual functioning. On the other hand, another study investigating 103 healthy individuals with frequent auditory verbal hallucinations described a lower level of functioning and higher scores on schizotypy compared to healthy controls (35). It could be argued that phenomenology of psychotic symptoms would probably differ between healthy subjects and patients, denoting distinct phenomena. But that appears not to be the case. An interesting study comparing voices heard by psychics to those heard by individuals with a psychosis diagnosis observed that psychotic experiences were rather similar in those two groups regarding the characteristics of the phenomena (36). Significant differences were only observed in age the voices started (psychics started hearing voices earlier in life), controllability of the phenomena, and voice’s contents (psychics’ voices had more often positive content, though negative content was not rare).

Regarding subclinical delusions, there seems to be a consensus regarding their association with lower level of functioning and higher level of psychopathology. For instance, a study in England with 7,281 subjects reported a prevalence of 18.6% of paranoid thinking in the year preceding subjects’ interview (37). People with paranoid thinking had poorer social functioning, higher stress at work, higher suicidal ideation, less happiness, and a greater presence of a range of psychiatric symptoms when compared to subjects without paranoid thinking.

In conclusion, psychotic symptoms are not exclusively manifested in schizophrenia and psychotic disorders. On the contrary, it’s a phenomenon widely distributed across other non-psychotic diagnoses, and across the general population with no psychiatric disorders as well. Psychotic phenomena are markers of severity in individuals with an established diagnosis. In people without a psychiatric disorder, psychotic phenomena may predict future diagnosis and is usually related to lower levels of functioning—although this may not happen for those with auditory hallucinations.

## Evidence of Distinct Biological Underpinnings for Positive Symptoms

Evidence supporting distinct biological pathways for positive symptoms in schizophrenia is less frequent, because it is generally investigated in connection with negative symptoms in subjects with the disorder. Since both domains generally are thought to co-occur, biological findings usually cannot be unquestionably ascertained to one or another symptom dimension. However, some recent reports suggest their biological distinctiveness—such as those comparing the biology of psychotic symptoms in schizophrenia and in non-psychotic diagnosis.

A study of familial aggregation of psychotic symptoms in BD pedigrees showed that there was a higher rate of psychotic symptoms among first-degree affectively ill relatives of probands (38). That is, BD patients with psychotic symptoms tended to have more relatives with psychotic symptoms than those BD individuals who had no psychotic symptoms. The authors suggest that psychotic BD may delineate a specific subtype worth investigating for singular genetic and biological traits. Another study reported on a cognitive signature of psychotic BD individuals (21). Those BD patients with psychotic symptoms were specifically impaired on measures of executive functioning and spatial working memory compared to those without psychotic symptoms. Patterns of findings suggested that psychotic symptoms may have neural correlates that are at least partially independent of those associated with BD more generally and more similar to those found in schizophrenia. Finally, psychotic BD patients were seen to have D2 dopamine receptor density changes, mimicking findings in individuals with schizophrenia (39).

Evidence of dopaminergic dysfunctions found in schizophrenia was also observed in other non-psychotic diagnoses. Altered dopamine transmission was described in psychotic symptoms of Alzheimer’s disease (40, 41), in posttraumatic stress disorder with psychotic features (42), and in psychotic depression (43). Other manifestations of the psychosis continuum were related to dopaminergic dysfunction too. Individuals in ultra-high risk for psychosis (UHR)—all of them meeting criteria for attenuated psychotic symptoms—were found to also have altered dopamine transmission (44, 45).

In schizophrenia, there is a consolidated knowledge about the involvement of dopamine in the pathophysiology of the disorder (46). This was mainly synthesized in the salience syndrome theory, which describes a dopamine-mediated attributional cognitive style of irrelevant stimuli (47). However, it wasn’t until recently that the salience syndrome was conceived to be “more a hypothesis of psychosis-in-schizophrenia. As such, it may have more implications for understanding the occurrence of psychosis in other illnesses (for example, manic psychosis) than it does for understanding the nonpsychotic (i.e., negative and cognitive) symptoms in schizophrenia” (47).

While positive symptoms are related to the temporal lobe and limbic areas (48, 49), contrastingly, negative symptoms in schizophrenia appear to be related to deficits in specific regions generating hypofrontality (50). Decreased frontal and prefrontal metabolism at rest during activation were found to be associated with negative symptoms in positron emission tomography (PET) (51, 52) and single photon emission computed tomography (SPECT) (53). Dopamine was also related to negative symptoms, yet in a different way compared to the D2 hyperactivity seen in psychosis (54, 55). Several studies described decreased levels of D1, D3, and D4 in the prefrontal cortex, possibly resulting in an inhibiting effect on behavior (56). A fewer number of studies implicate serotonin dysfunction in the pathophysiology of negative functions (57, 58), with one report specifically describing decreased serotonin binding in the amygdala (59). As for the acetyl-choline neurotransmission, several authors have reported that patients with schizophrenia with lower β2-nicotinic acetylcholine receptor availability had greater negative symptoms (60). This finding was also consistent with the observation that in some studies, the heaviest smokers with schizophrenia had the lowest severity of negative symptoms (60, 61). More importantly, it has been recently considered that a reduction in glutamate signaling in the brain would play a key role in negative symptoms (62). This has been suggested by clinical trials targeting N-methyl-D-aspartate (NMDA) receptors for the improvement of negative symptoms (63) and by neuroimaging and postmortem studies addressing glutamate transmission and NMDA receptors (64).

As such, the latest theories regard positive symptoms as biologically independent features of schizophrenia, recruiting different pathways compared to other symptom dimensions. Negative symptoms, as well as cognitive symptoms, result from changes in multiple transmitter/neural system that would precede the onset of psychosis (65). Only in a late stage would these pathways lead to dopamine hyperfunction, triggering the apparent clinical psychosis and leading to the assignment of the schizophrenia label (**Table 1**) (47, 55).

**Table 1:** Main characteristics of positive symptoms versus negative symptoms.

Positive symptoms	Negative symptoms*
**Unspecific: Present in the general population (continuum) and in psychiatric diagnosis at large, being a marker of severity**	Present to a minor extent in some psychiatric diagnosis; far more related to schizophrenia
**Dopaminergic (D2) dysfunction observed all accross the phenotipic continuum of psychosis**	Serotonine, acetyl-choline, and mainly glutamate dysfunction. May affect dopamine receptors (D1, D3, D4)
**Temporal lobe, limbic areas**	Frontal and prefrontal cortex
**Amenable to adaptation, generally responsive to antipsychotics**	Insidious onset, enduring, do not respond to medication, generates disability
**More evident, draw physician’s and public attention, related to stigma**	Furtive, may progress unnoted, less related to stigma
**Generally do not predict outcome in schizophrenia**	Related to worse outcome in schizophrenia

*Primary negative symptoms, not including secondary negative symptoms.

## Reframing Schizophrenia

Summarizing, hallucinations and delusions are pervasive, non-specific phenomena. They are associated with other non-psychotic diagnosis and are present in healthy individuals of the general population as well. Pervasiveness is further reinforced by biological findings, which suggest that positive symptoms are independently underpinned from other symptom dimensions. Dopamine dysfunction is consistently found in schizophrenia as well as in psychotic features of non-psychotic diagnosis, and in other expressions of the psychosis continuum. Finally, some authors argue that dopamine dysregulation would be a theory of “psychosis-in-schizophrenia” rather than a theory for understanding schizophrenia in its entirety (55).

So why should we conceive non-specific symptoms such as positive symptoms as core characteristics in schizophrenia, using them for diagnostic purposes and to drive research? Here we resume Canstatt’s idea at the very beginning of psychosis’ conception: shouldn’t psychotic symptoms be considered non-specific proxies for brain suffering in schizophrenia—as they are in other disorders like depression, BD, or Alzheimer’s disease?

In fact, resembling Bleuler, in a recent past, other symptom dimensions of today’s “schizophrenia” were already regarded as definers of a more robust disease model. The concept of deficit schizophrenia (DS) was introduced by Carpenter et al. in 1988 to describe patients showing primary and enduring negative symptoms (66). Subjects with DS would have poorer premorbid adjustment, which would start early in life and be present in all the individual’s developmental stages (67). Besides, DS would also be related to a longer duration of untreated psychosis (68). According to the authors, DS would configure a more consistent and homogenic disease entity within the schizophrenia syndromes.

A similar concept was later popularized by Murray et al. (69) in 1993, videlicet, the neurodevelopmental theory of schizophrenia. For decades, there was a debate on whether deterioration in schizophrenia would be secondary to psychosis—the neurodegenerative hypothesis—or if it would begin before the first psychotic outbreak—the neurodevelopmental hypothesis (70). It recently became clear that cognitive and functional loss, accompanied by correlated structural brain changes, would start years before the emergence of first episode psychosis (65).

Indeed, the degenerative process occurring before the first psychotic outbreak has deep implications in outcome. In an investigation about the course of schizophrenia published by Brill et al. (71), premorbid intelligence and behavioral functioning directly predicted postmorbid negative symptoms and indirectly predicted postmorbid social and occupational symptoms, *via* negative symptoms. On the other hand, positive symptoms were not significantly associated with functional outcomes. Another work conducted by Addington and Addington described that poor premorbid functioning and poor outcome were significantly associated with negative symptoms (72). Bailer et al. showed that in 163 individuals with schizophrenia, premorbid adjustment was significantly associated with negative symptoms and social disability over the 3-year course of the illness (73). Strous et al. analyzed 111 individuals with schizophrenia and described a progressively poor premorbid functioning before the onset of the disease, relating this factor to poorer outcomes (74). All these authors suggested that schizophrenia would start long before the first psychotic episode outbreak, an idea that was presently confirmed by the established acknowledgment of a prodromal psychosis field of research (75).

Hence, in a great set of cases of “schizophrenia,” we only intercept the disease’s course when it’s far too late—i.e., by the time the first psychotic episode elapses with its rampant positive symptoms, when outcome is already somewhat outlined. It seems that, on the occasion of the first psychotic outbreak, we face at least two pathological processes, with distinct courses: A) Having good premorbid functioning and a low level—or absence—of negative symptoms shows a disease process that has barely—or not even—started. As such, positive symptoms are clinically and pathologically central aspects, and good outcomes are expected. Chance of full recovery is augmented, increasing the odds of considering it a brief psychotic episode, a “schizophreniform” disorder, or a single (or few) episode “schizophrenia” with good outcome—in other words, a non-DS. In case of persistence of positive symptoms, the disorder may alternatively fall into the delusional disorder, or the chronic hallucinatory psychosis categories, for instance. B) However, if the first psychotic outbreak emerges in a premorbid progressively poorer overall functioning scenario—regarded consequently as core aspects—it most probably denotes a long-time ongoing subclinical disease/prodrome (76). One would expect worse outcomes, as a declining course resembling that of Kraepelin’s *dementia praecox*, the need of social and cognitive rehabilitation, worse antipsychotic response. Such individuals would most probably be diagnosed as having schizophrenia—or DS, alternatively (7, 77).

Recollecting the original meaning of schizophrenia discussed at the beginning of this work, first Kraepelin conceived it as psychotic symptoms plus cognitive decline under the term *dementia praecox*. Then came Bleuler and refined the concept, setting the 4 A’s—cognitive and negative symptoms—as core disturbances in schizophrenia, relegating today’s psychotic symptoms to the second rank. This reframing would thus make sense with the original schizophrenia concept. But it’s not a mere question of conceptualization or semantics, for this new (but old) schizophrenia would make more sense biologically as a mind’s disease.

Consequently, contrasting the original meaning of the term with today’s concept creates an apparent incoherence, because we use positive symptoms to diagnose “schizophrenia,” while in schizophrenia, what is really important in terms of diagnosis and prognosis is the individual’s premorbid level of non-psychotic symptoms and functioning, which will endure and probably get worse after the first psychotic episode.

More importantly, in fact, subtly progressing negative/cognitive symptoms and deteriorating functioning should dictate our disease concept of schizophrenia instead of positive symptoms, reclaiming Bleuler’s original conceptualization. For the first, predict disability—an essential feature of psychiatric diagnosing—over time, and not the latter (78).

**Figure 1:**
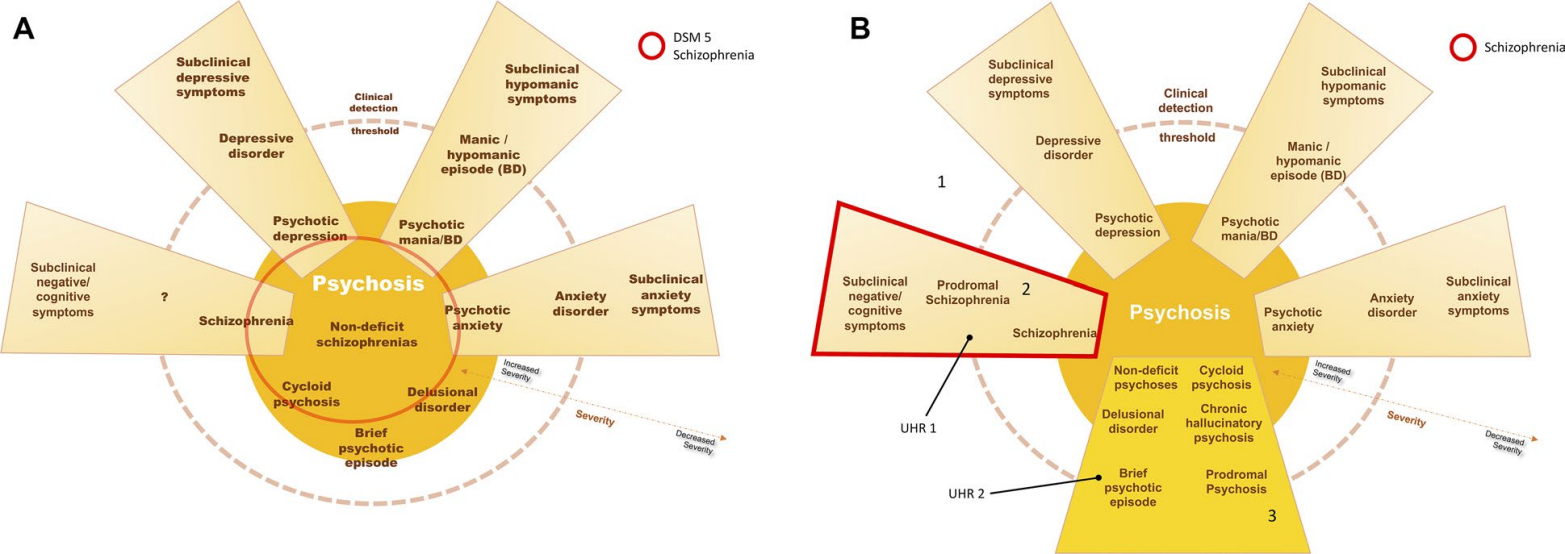
In **(A)**, current schizophrenia is represented, including several entities with positive symptoms, without a biological underpinning for the diagnosis. In **(B)**, schizophrenia is represented by the schizophrenia continuum, 2, in which negative and cognitive deficits are core aspects of the disorder–neurodevelopmental psychosis. Area 3 represents other psychotic processes in which positive symptoms are core aspects instead—including the concepts of non-deficit schizophrenia and neurodegenerative psychosis. Accordingly, individuals at ultra-high risk for psychosis would be distinguished between those with cognitive decline and subtle negative symptoms (UHR 1—worst outcome) and those without such deterioration (UHR 2—more benign outcome). Area 1 would represent the subclinical symptom spectrum, with increased interchange and blending between symptom dimensions. (Blending of dimensions, e.g., schizoaffective disorder, is not represented in this scheme).

As such, schizophrenia should be considered a cognitive and negative symptom—a neurodevelopmental—disorder instead of a primary psychotic disorder (**Figure 1**). Only in the absence of such premorbid deficits should psychosis be considered as the disease *per se* (primary dopaminergic imbalance): brief psychotic episode, schizophreniform disorder, delusional disorder, and chronic hallucinatory psychosis. Non-deficit “schizophrenia,” for instance, should be dispersed across these psychosis diagnoses, which should include Leonhard’s cycloid psychosis, for instance (79). Such primary psychotic disorders have distinct courses, biological underpinnings, and may have good outcomes, for positive symptoms are more likely to cease with antipsychotics and are more amenable to adaptation—while even normal people with psychotic phenomena can live fully adapted and unmedicated, as discussed previously.

However, hallucinations and delusions emerging in a premorbid deficit scenario—secondary dopaminergic imbalance—should be considered as a late-stage epiphenomenon of a neurodevelopmental schizophrenia process—NMDA-mediated hypofrontality—started long before the appearance of the positive symptoms. Psychosis would be only a proxy for severe brain stress—reclaiming its first meaning by Canstatt—the manifestation of a long-standing toxic process’s apex. This underlying process is insidious, enduring, treatment resistant, and disabling (**Table 2**).

**Table 2:** Sketch of syndromal diagnostic criteria for schizophrenia versus psychotic disorder.

Schizophrenia spectrum	Psychotic disorder spectrum
**Subclinical phase**	- Syndrome characterized by acute psychotic symptoms—hallucinations or delusions—not preceded by over a year of cognitive decline and/or negative symptoms.
**- Presence of subtle, persistent cognitive decline starting in infancy and early adulthood**	- Can be characterized by disorganized speech or behavior, if no previous progressive impairment is observed
**- Unobtrusive progression of negative symptoms starting in infancy and early adulthood**	- Include: delusional disorder, cycloid psychosis (that would cover some types of schizophreniform disorders, and schizophrenias with full recovery and no cognitive decline or negative symptom progression before the first episode psychosis), brief psychotic episode.
**Prodromal phase** **- Unspecific depressive and/or anxiety symptoms, starting some years after the subclinical phase, and lasting 1 or 2 years**	- Exclude: mood disorders with psychotic features, substance-induced psychotic episode, psychotic symptoms due to a somatic condition.
***Psychotic phase*** **- Irruption of psychotic symptoms following the prodromal phase** **May be absent in ** ***Schizophrenia Simplex***	
– Exclude: neurodevelopmental disorders, neurocognitive disorders, and substance use disorders, as primary cause of early cognitive decline; psychotic disorders and mood disorders, due to different syndromal presentation.	

As such, this is the pathology—represented by pre-psychosis subtle cognitive decline and increasing negative symptoms—that should be more intensely addressed by research and preventative medicine under the label of schizophrenia—as Bleuler first suggested it.

## Implications

There are several implications in considering schizophrenia as a primary negative/cognitive dysfunction.

The first issue concerns diagnostic validity. The previously discussed concept of DS wished to identify a relatively homogeneous subgroup of individuals with primary and enduring negative symptoms. According to authors (66), it contrasts those individuals with secondary and phasic negative symptoms, the result of identifiable sources such as positive symptoms, antipsychotic treatment, or social isolation. Indeed, numerous findings of distinct biological correlates corroborate this division (80), observing that DS and non-DS have differences in genetic, neurocognitive, electrophysiological, and brain imaging findings (80, 81). More importantly, both forms of “schizophrenia” also differ in treatment response (82). Ultimately, the DS and non-DS divisions also reinforce the poor distinctiveness capability of positive symptoms, for severity of hallucinations and delusions are comparable in both categories (83, 84). Actually, the present conceptual shift suggestion partly resumes the DS versus non-DS distinction, as schizophrenia would somewhat reflect the DS concept, while non-DS should be dissolved and put together with other primary psychosis diagnosis.

Unlike the original DS discussion, which focused mainly on assessments after the first episode psychosis, the present reframed schizophrenia concept would outline a disorder starting subtly years before the outbreak of positive symptoms. Refining the concept of schizophrenia as a disorder with negative and cognitive symptoms as its core should better drive research towards the biology underlying the schizophrenic process, as it was proposed with DS. This reframed concept of schizophrenia would have increased validity (85), as shown by a) antecedent validity—familial aggregation of negative symptoms (86), its relation to primary and enduring negative symptoms in schizophrenia (87), and strong genetic component (88, 89); b) concurrent validity—biological findings discussed before (90) and independent biological underpinnings (56); and c) predictive validity—worse outcome described above (91).

Second, and consequently, schizophrenia, being mainly defined by subtle cognitive deterioration and negative symptoms rather than a syndrome encompassing heterogeneous-sourced hallucinations and delusions, would be a more reliable disease entity. Positive symptoms are apparent to the physician and to the public, but they are like fever. In medicine, fever is usually taught under the “febrile syndromes” denomination, where we are trained to investigate what the underlying causes of fever are. Fever can be a result of acute processes—pneumonia, flu, and meningitis—as well as of chronic processes—tuberculosis and leukemia. Fever can also be a result of endogenous dysregulations, such as those found under the periodic fever syndromes. Analogously, psychotic symptoms would be found in acute processes—psychotic depression, manic depression, and *delirium*—as well as in chronic processes—Alzheimer’s disease and schizophrenia. Psychotic symptoms could also be the result of endogenous dysregulations, such as delusional disorder, brief psychotic episode, cycloid psychosis, chronic hallucinatory disorder, etc. Fever is observed by the physician, and diagnoses of tuberculosis or AIDS, for instance, are confirmed by biological tests. In the case of AIDS, HIV infection can be detected in symptom-free individuals, which can present with asymptomatic low levels of CD4 for years. Likewise, in schizophrenia cognitive tests, neuroimaging, inflammatory, and other biological tests, along with careful negative symptom and social and general functioning examination, should detect the subclinical/asymptomatic disease process—“infection”—before the first outbreak of psychosis—“AIDS”—emerges. This framework should definitely put schizophrenia under the disease entity conceptualization, Mimicking other diagnoses in medicine. Following current diagnostic manuals and remaining in the phenomenic stratum would be like facing fever as a disease and not as a consequence of one.

Third, hallucinations and delusions typically represent a mismatch between the subject and reality. The incongruity evoked by such manifestations deepen the public discriminative process of separating “us” from “them” (92), leaving individuals with these symptoms more vulnerable to stigma (93). As such, most of the stigmatic burden of “schizophrenia” results from the expression of positive symptoms—they are more evident to the general population, they draw more attention, and so they attract more public stigma. Moreover, this link between stigma and hallucinations/delusions is not new, for it represents a century-old tradition of discriminating “madness”. People with such manifestations were always referred to as lunatics, madmen, bedlamites, etc. The link between positive symptoms, madness, and stigma is deep-rooted and persistent, being represented in the current days by the burden attached to the schizophrenia label (94, 95). Disheveling positive symptoms from the schizophrenia concept should partly reduce stigma towards schizophrenia, diminishing treatment avoidance due to fear of labeling and discrimination. On the other hand, framing positive symptoms as a non-specific dimensional manifestation, occurring also in healthy and adapted individuals, should also help to break the bond between “madness” and stigma.

## Caveats

Some caveats surround the current proposal. The existence of a specific disease course would contradict this conceptualization, namely, an acute psychosis onset without a full recovery—with marked and enduring negative and cognitive symptoms after the first psychotic episode. A review study observed prevalence rates of such courses of 7–23% (7). In the face of these residual symptoms, it could be argued that this would represent either one of two cases: a) acute onset was not really acute, and a subclinical pathological process would have been missed, being detectable well in advance by more accurate tests other than clinical evaluation–neurocognitive tests, for instance; or b) such acute episodes would last longer than usual or would be more severe than others, resulting in increased brain toxicity, which would leave negative and cognitive symptoms as sequels in the post-crisis phase. This hypothesis is reinforced by studies relating longer duration of untreated psychosis to a worse outcome (96). It also takes side with the neurodegenerative hypothesis, highlighting the noxious effect of untreated psychotic symptoms (97). At last, one cannot suppose that a perfectly adapted and healthy individual—with no premorbid signs—with a first episode psychosis, having his/her episode adequately treated to keep it brief, would evolve to a DS, contradicting the present framework. However, the possibility of this specific course should be further investigated.

The second caveat concerns the yet heterogeneous aspect of considering schizophrenia as a negative and cognitive syndrome. Several studies have reported weak relationships between cognitive dysfunctions and negative symptoms, but overall evidence confirmed that the two domains are biologically independent (98, 99). Relegating psychotic symptoms to a second plan in schizophrenia would increase validity and improve homogeneity, but further future steps should still be taken. Several authors propose that there would be different syndromes within the negative dimension of schizophrenia (100), and this hypothesis should be further biologically investigated.

At last, due to space restrictions and to maintain focus, the present work does not engage the delimitation to other psychiatric disorders, limiting itself to point psychotic symptoms as a marker of severity. Setting schizophrenia as an “acquired” neurodevelopmental disease—in contrast to autism, e.g., an innate neurodevelopmental disease—would increase the disease entity’s homogeneity, allowing for better biological understanding of it. Future directions would also analyze the discriminatory power of specific symptoms and syndromes, which is not approached here.

## Conclusion

The present work proposes a conceptual shift of schizophrenia from a psychotic disorder to a cognitive and negative symptom disorder. Individuals nowadays diagnosed with “schizophrenia” without premorbid cognitive/negative deficits would be re-diagnosed to other psychotic diagnoses—brief psychotic episode, cycloid psychosis, chronic hallucinatory disorder, etc. Only those with a premorbid history of increasing cognitive/negative deficits would be diagnosed as having schizophrenia. This should occur regardless of the presence of a first psychotic outbreak, enabling very early intervention, acknowledging that some deteriorative processes might be interrupted with effective preventative interventions before first episode psychosis and, on the other hand, also allowing the conceptual existence of *schizophrenia simplex*.

Such shift should increase validity of a schizophrenia diagnosis by better outlining it as a disease entity and consequently approximating it to the medical model of disease (instead of the “disorder” model), better drive research to understand the biological underpinnings of schizophrenia in order to improve primary prevention and treatment, and potentially diminish stigma by uncoupling it from positive symptoms and from the schizophrenia concept.

## Author Contributions

AL conducted literature review and preparation of the manuscript.

## Conflict of Interest Statement

The authors declare that the research was conducted in the absence of any commercial or financial relationships that could be construed as a potential conflict of interest.
